# Identification of a Ubiquitin Related Genes Signature for Predicting Prognosis of Prostate Cancer

**DOI:** 10.3389/fgene.2021.778503

**Published:** 2022-01-17

**Authors:** Guoda Song, Yucong Zhang, Hao Li, Zhuo Liu, Wen Song, Rui Li, Chao Wei, Tao Wang, Jihong Liu, Xiaming Liu

**Affiliations:** ^1^ Department of Urology, Tongji Hospital, Tongji Medical College, Huazhong University of Science and Technology, Wuhan, China; ^2^ Second Clinical College, Tongji Medical College, Huazhong University of Science and Technology, Wuhan, China; ^3^ Department of Geriatrics, Tongji Hospital, Tongji Medical College, Huazhong University of Science and Technology, Wuhan, China

**Keywords:** prostate cancer, ubiquitin, prognostic signature, prognosis, bioinformatics

## Abstract

**Background:** Ubiquitin and ubiquitin-like (UB/UBL) conjugations are one of the most important post-translational modifications and involve in the occurrence of cancers. However, the biological function and clinical significance of ubiquitin related genes (URGs) in prostate cancer (PCa) are still unclear.

**Methods:** The transcriptome data and clinicopathological data were downloaded from The Cancer Genome Atlas (TCGA), which was served as training cohort. The GSE21034 dataset was used to validate. The two datasets were removed batch effects and normalized using the “sva” R package. Univariate Cox, LASSO Cox, and multivariate Cox regression were performed to identify a URGs prognostic signature. Then Kaplan-Meier curve and receiver operating characteristic (ROC) curve analyses were used to evaluate the performance of the URGs signature. Thereafter, a nomogram was constructed and evaluated.

**Results:** A six-URGs signature was established to predict biochemical recurrence (BCR) of PCa, which included ARIH2, FBXO6, GNB4, HECW2, LZTR1 and RNF185. Kaplan-Meier curve and ROC curve analyses revealed good performance of the prognostic signature in both training cohort and validation cohort. Univariate and multivariate Cox analyses showed the signature was an independent prognostic factor for BCR of PCa in training cohort. Then a nomogram based on the URGs signature and clinicopathological factors was established and showed an accurate prediction for prognosis in PCa.

**Conclusion:** Our study established a URGs prognostic signature and constructed a nomogram to predict the BCR of PCa. This study could help with individualized treatment and identify PCa patients with high BCR risks.

## Introduction

Prostate cancer (PCa) is one of the most common malignancies worldwide with the third highest cancer-causing deaths following lung cancer and colorectal cancer in American males (Schatten, 2018). The curative therapies for primary tumors are radical prostatectomy or radiation therapy (Mateo et al., 2019). Nearly one third patients would suffer biochemical recurrence (BCR) at 10 years after radical prostatectomy without neoadjuvant or adjuvant therapy (Liesenfeld et al., 2017). Without further treatment, the median time from BCR to metastasis and from metastasis to death is 8 and 5 years, respectively (Freedland et al., 2005). Thus, it is important to identify the patients with high risk of BCR.

Ubiquitin and ubiquitin-like (UB/UBL) conjugations are vital post-translational modifications which participate in nearly all biological processes and pathways such as protein degradation and turnover, intercellular signal transduction, cell cycle and DNA damage repair (Swatek and Komander, 2016). Ubiquitin is a highly conserved heat-stable protein with 76 amino acids. The process of ubiquitin conjugation is a successive three-step cascade which is catalyzed by three enzymes including ubiquitin-activating enzymes (E1s), ubiquitin-conjugating enzymes (E2s), and ubiquitin protein ligases (E3s) (Pickart, 2001). However, deubiquitinating enzymes (DUBs) remove Ub or UBL moieties from protein and reverse the ubiquitination process (Heride et al., 2014). In addition, the protein containing ubiquitin-binding domain (UBDs) and ubiquitin-like domains (ULDs) also plays an important role in regulating the ubiquitination process (Buchberger, 2002; Heride et al., 2014). Studies have revealed that the dysfunction of protein ubiquitination would be involved in many human pathologies such as tumorigenesis, and neurodegeneration (Popovic et al., 2014). However, no studies have investigated the association between URGs and the prognosis of PCa patients.

In this study, we downloaded transcriptome data and clinicopathological data from the Cancer Genome Atlas (TCGA) and Gene Expression Omnibus (GEO) databases, and performed bioinformatics analysis to identify a URGs signature to predict the prognosis of PCa patients.

## Materials and Methods

### Data Collection and Processing

The transcriptome data and corresponding clinicopathological data, containing 499 tumor and 52 adjacent normal tissues, were downloaded from TCGA (https://portal.gdc.cancer.gov/). The transcriptome data has been background adjusted and normalized with the style of fragments per kilobase million (FPKM) (Mortazavi et al., 2008). Normalized mRNA expression data from GSE21034 dataset, which was served as a validation cohort, were downloaded from GEO database. The genes would be deleted if their expression values were 0 in more than 50% samples. Average expression values were evaluated if genes were duplicated. Then, nine E1s, 43 E2s and 900 E3s were identified from the iUUCD 2.0 database (http://iuucd.biocuckoo.org/). 952 URGs were identified.

### Construction and Evaluation of URGs Prognostic Signature for BCR

The expression data of 902 URGs were extracted from processed transcriptome data of TCGA dataset. The batch effect and unwanted variation were removed using “sva” R package between the two datasets. The univariate Cox regression analysis was performed to calculate the association between URGs and BCR-free survival. Next, the least absolute shrinkage and selection operator (LASSO) Cox regression analysis was used to select the valuable prognostic URGs by using the “glmnet” package in R software (Version 4.1.0). Then, a stepwise multivariate Cox regression proportional hazards regression model was established to further select URGs and optimize the model. Finally, a risk score formula was constructed based on the regression coefficients of multivariate Cox analysis and the expression values of corresponding URGs. The risk score formula was listed: *Risk score = (exp Gene1 ×coef Gene1) + (exp Gene2 × coef Gene2) +…+ (exp GeneN × coef GeneN)*. Here, exp represents the expression of optimized genes, and coef represents estimated multivariate Cox regression coefficients.

Both median value and optimal cut-off value are widely used for the stratification in survival analysis. Here, we used median risk score of training dataset as the same cut-off value for both training and validation datasets. Kaplan-Meier curve analysis (using “survival” package) and the area (AUC) under receiver operating characteristics (ROC) curve analysis (using the “timeROC” package) were performed to investigate the predictive value of the URGs-based signature. Moreover, GSE21034 dataset was served as a validation set to verify the stability and accuracy of URGs signature. *p* values < 0.05 were considered as statistical significance.

### Correlation Between Prognostic Signature and Clinicopathological Parameters

To investigate the clinical significance of the URGs signature, the patients from TCGA were stratified by clinicopathological parameters containing age, pathological T stage (pT), Gleason grade score (GGS), surgical margin status (SMS), and prostate specific antigen (PSA). Kaplan-Meier curve analysis was used to investigate the prognostic value of the signature in different subgroups. In addition, we assessed the URGs signature risk score distribution according to different clinicopathological variables. *p* values < 0.05 were considered as statistical significance.

### Construction and Validation of a Nomogram

Univariate Cox analysis and multivariate Cox analysis were performed to identify independent prognostic parameters combined the URGs signature and clinicopathological parameters in TCGA dataset. Next, a prognostic nomogram was constructed to predict 1-, 3-, and 5-year BCR-free survival for PCa patients. Calibration plots were used to evaluate the reliability of the nomogram. Then, Kaplan-Meier curve analysis, the AUC under ROC curve, and C index (using the “survcomp” package) were performed to evaluate the performance of the nomogram. *p* values < 0.05 were considered as statistical significance.

## Results

### Construction and Validation of URGs Prognostic Signature

**FIGURE 1:**
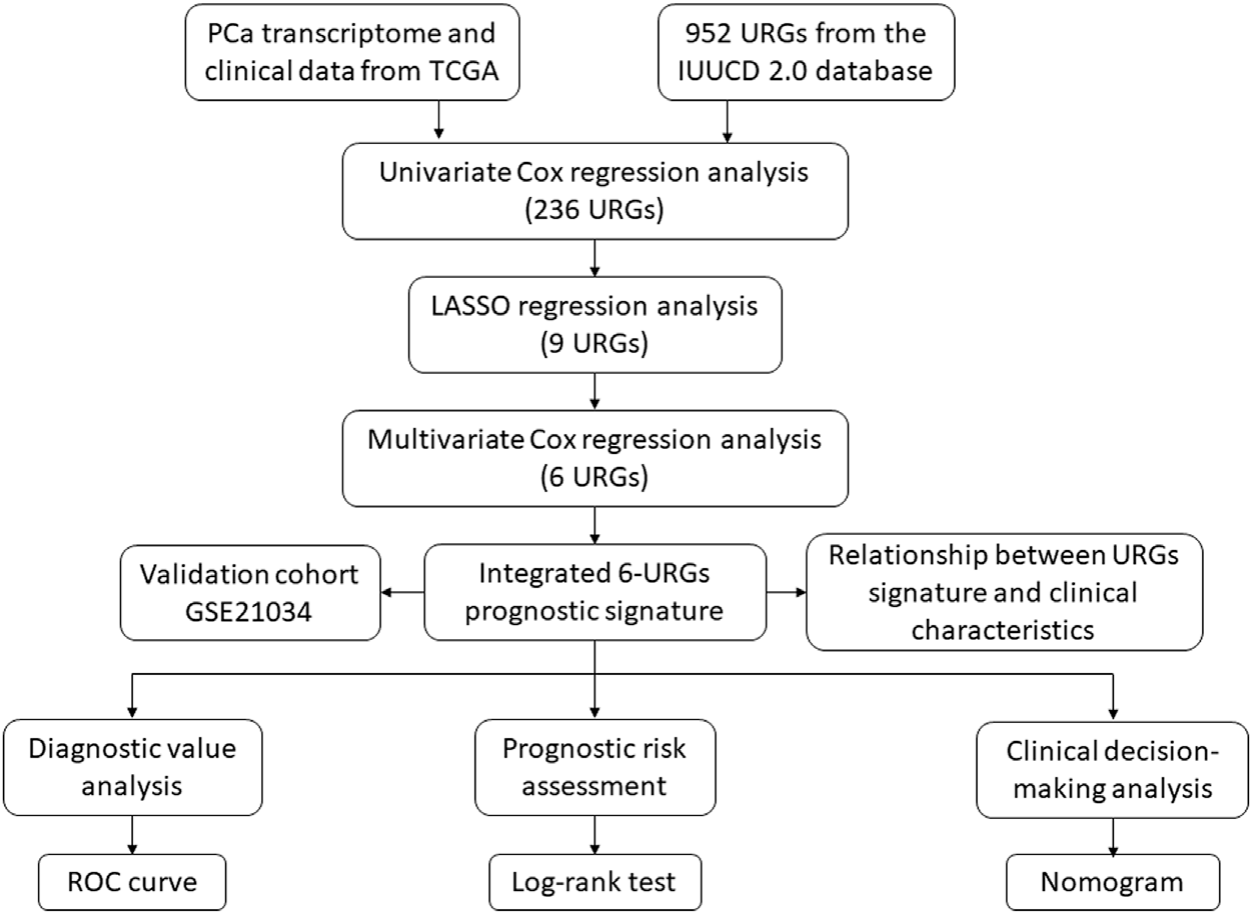
The flowchart of the study procedures. PCa, prostate cancer; TCGA, the Cancer Genome Atlas; URGs, ubiquitin related genes; LASSO, the least absolute shrinkage and selection operator; ROC, receiver operating characteristic.

**FIGURE 2:**
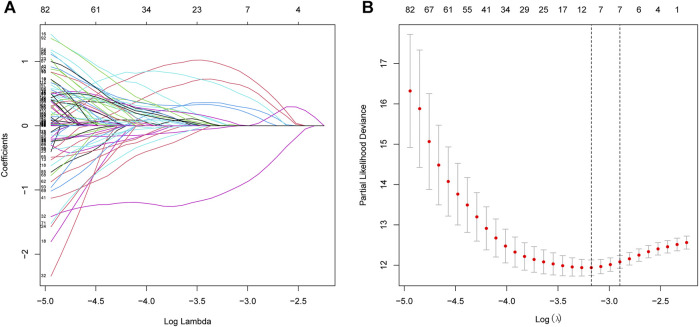
Selection of prognostic URGs by LASSO regression. **(A)** LASSO coefficient profiles of the prognostic genes. **(B)** Parameter selection in LASSO model. URGs, ubiquitin related genes; LASSO, the least absolute shrinkage and selection operator.

**FIGURE 3:**
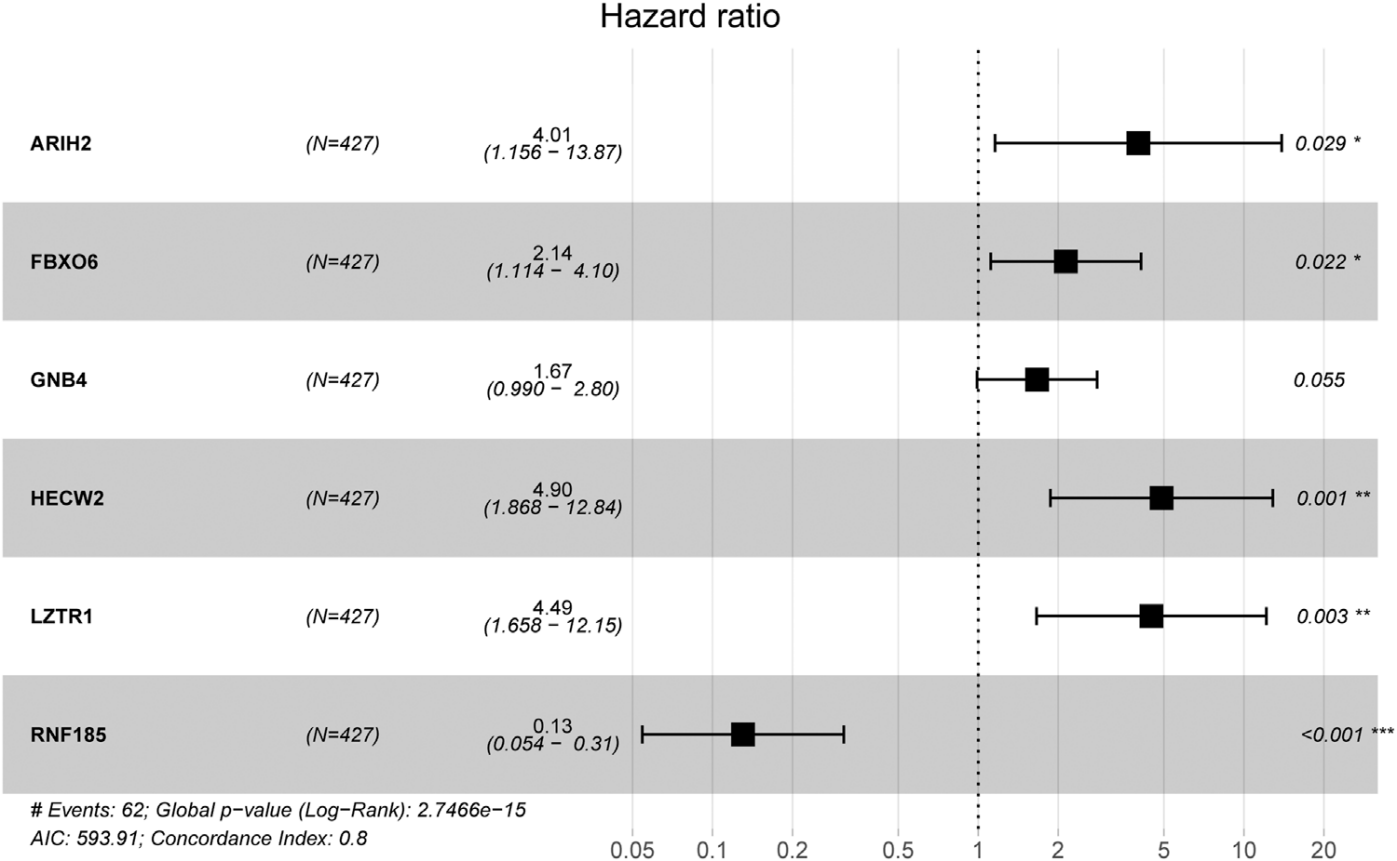
Multivariate Cox regression analysis to identify prognostic genes.

The procedure of this study is listed in Figure 1. BCR information and transcriptome data of 427 patients were obtained from the TCGA database. The univariate Cox regression analysis found that the expression of 236 URGs were significantly correlated with BCR prognosis of PCa patients (*p* < 0.05; Supplementary Table S1). LASSO Cox regression analysis was then applied for further analysis, and nine URGs were identified (Figure 2). Subsequently, a six URGs based prognostic signature was established by performing multivariate Cox regression analysis (Figure 3). The risk scores were calculated by following formula: *Risk score = (1.3876×ARIH2exp)+(0.7596×FBXO6exp)+(0.5102×GNB4exp)+(1.5888×HECW2exp)+(1.5015×LZTR1exp)+(−2.0379×RNF185exp).*

**FIGURE 4:**
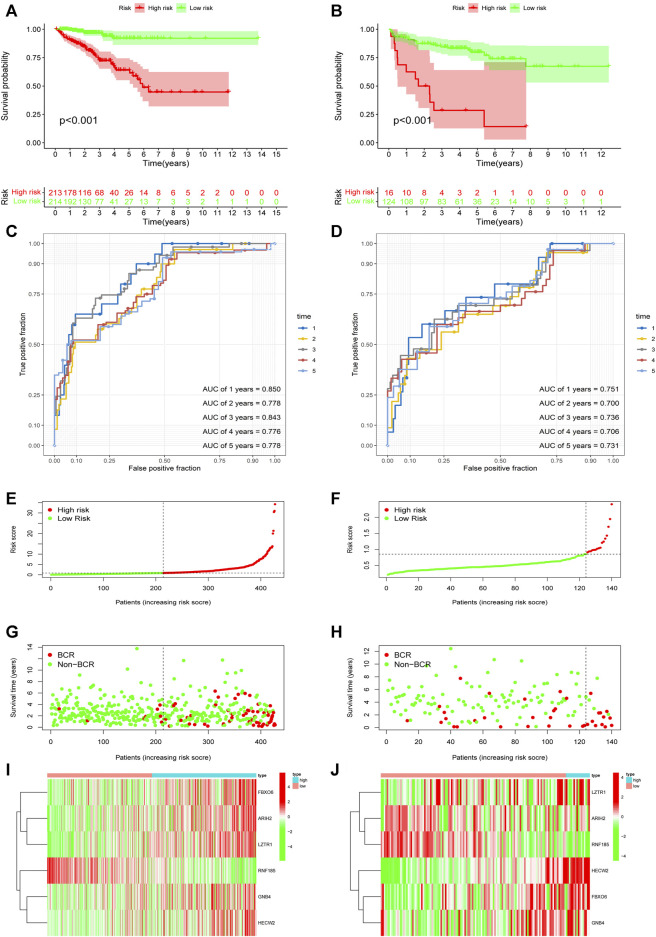
Evaluation and validation of the predictive value of URGs signature in the TCGA dataset and GSE21034 dataset. **(A)** Kaplan-Meier curve analysis between high-risk and low-risk subgroups in the TCGA dataset. The subgroups were stratified by the optimal cut-off value for the risk scores. **(B)** Kaplan-Meier curve analysis between high-risk and low-risk subgroups in the GSE21034 dataset. The subgroups were stratified by the optimal cut-off value for the risk scores. **(C)** The AUCs under ROC for first, second, third, fourth and fifth year BCR predictions based on URGs signature in the TCGA dataset. **(D)** The AUCs under ROC for first, second, third, fourth and fifth year BCR predictions based on URGs signature in GSE21034 dataset. **(E**, **G**, **I)** The distribution of survival status and risk score, and heat map of prognostic genes expression in the TCGA dataset. **(F**, **H**, **J)** The distribution of survival status and risk score, and heat map of prognostic genes expression in the GSE21034 dataset. URGs, ubiquitin related genes; TCGA, the Cancer Genome Atlas; AUC, area under ROC curve; ROC, receiver operating characteristic; BCR, biochemical recurrence.

Based on the URGs signature, patients were divided into high-risk and low-risk subgroups according to the same cut-off value in TCGA dataset and GSE21034 dataset. In the TCGA dataset, Kaplan-Meier curve analysis showed that the patients in the high-risk group had a poorer BCR-free survival prognosis than those in low-risk group (Figure 4A). AUC values of different time point were estimated and the results showed that the AUC values were 0.850 at first year, 0.778 at second year, 0.843 at third year, 0.776 at fourth year and 0.778 at fifth year. It indicated that this signature had a good prognostic predictability (Figure 4C). Then, GSE21034 dataset was used to verify the performance of the URGs signature. The result of Kaplan-Meier curve analysis also revealed that the BCR-free survival prognosis of patients in high-risk group was poorer than those in low-risk group (Figure 4B). The AUC values were 0.751, 0.700, 0.736, 0.706 and 0.731 at first, second, third, fourth and fifth year, respectively (Figure 4D). The distribution of risk score, recurrence status and gene expression heat maps were showed in Figures 4E–J.

### Association Between Prognostic Signature and Clinicopathological Parameters

**FIGURE 5:**
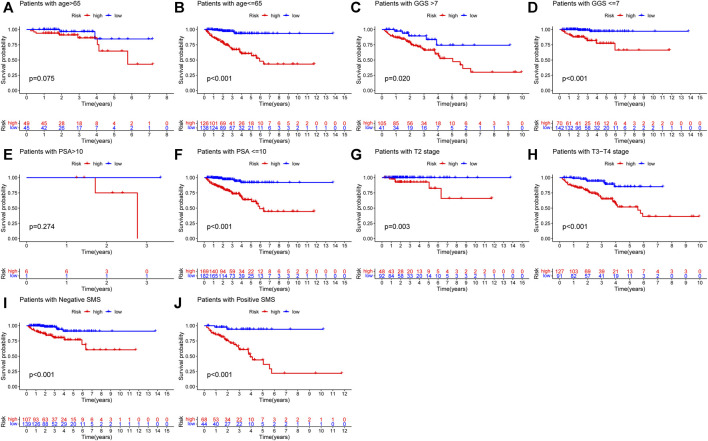
Kaplan-Meier curve analysis of PCa patients in different clinicopathological stratifications in TCGA dataset. **(A)** Age>65. **(B)** Age ≤ 65. **(C)** Gleason score>7. **(D)** Gleason score ≤ 7. **(E)** PSA value >10. **(F)** PSA value <=10. **(G)** Pathological T stage: T2. **(H)** Pathological T stage: T3-T4. **(I)** Negative SMS. **(J)** Positive SMS. PCa, prostate cancer; PSA, prostate specific antigen; SMS, surgical margin status.

In order to explore the association between the URGs signature and clinicopathological parameters, we stratified patients in TCGA dataset according to age, GGS, PSA, pT, and SMS. Then, Kaplan-Meier curve analysis was performed and the results showed that high-risk patients had poorer BCR-free survival prognosis compared to low-risk patients in all stratifications except for patients with age >65 and PSA >10 (Figure 5).

**FIGURE 6:**
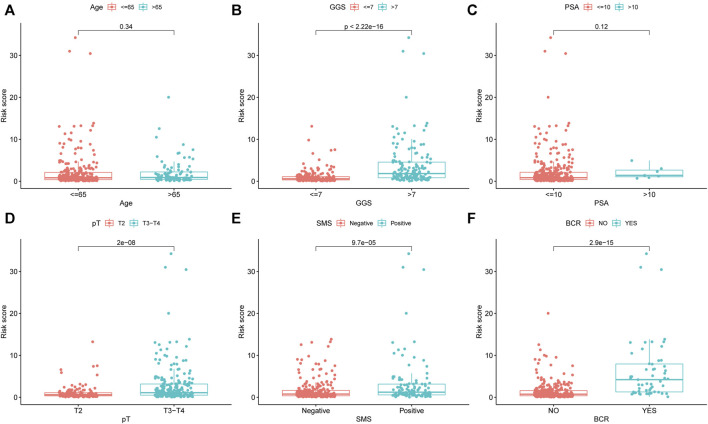
The differential distribution of URGs signature risk scores between different clinicopathological variables and survival status in the TCGA dataset. **(A)** Age. **(B)** GGS. **(C)** PSA. **(D)** pT. **(E)** SMS. **(F)** BCR. URGs: ubiquitin related genes; GGS, Gleason grade score; PSA, prostate specific antigen; pT, pathological T stage; SMS, surgical margin status; BCR, biochemical recurrence.

In addition, we compared the risk score distribution in different clinicopathological stratifications to investigate the association between prognostic signature and the tumor clinical characteristics. The results showed that the patients with higher GGS, higher PSA, higher pT, and positive SMS had higher URGs signature risk scores (Figures 6B,D,E). The patients with BCR also had higher risk scores (Figure 6F). However, no significant difference existed between the subgroups stratified by age and PSA (Figures 6A,C).

### Construction and Validation of a Nomogram

**FIGURE 7:**
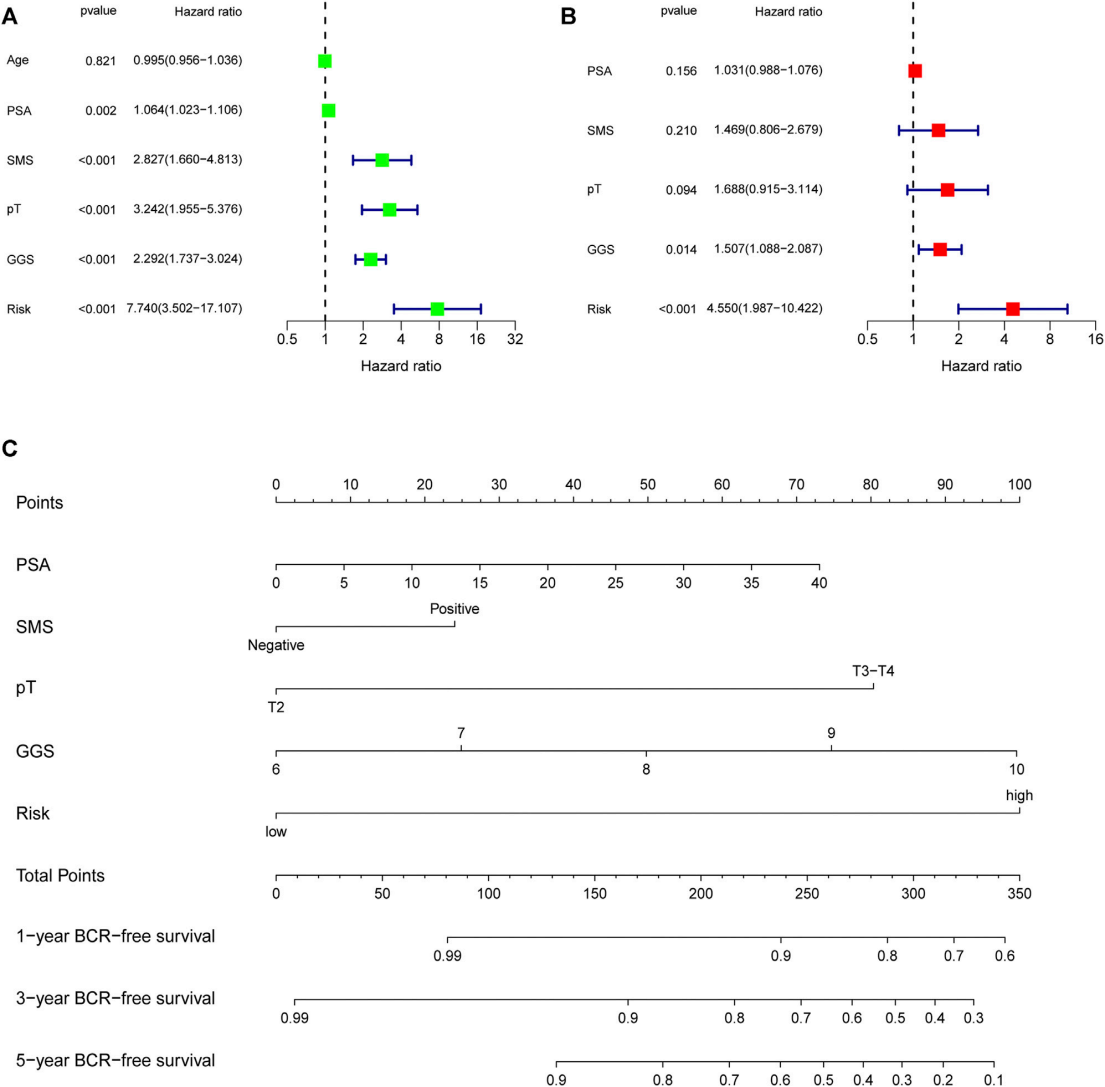
Evaluation of the independent prognostic parameters based on clinicopathological variables and the signature, and construction of a nomogram in the TCGA dataset. **(A)** Univariate Cox analysis and **(B)** multivariate Cox analysis for evaluating independent prognostic factors. **(C)** Nomogram for predicting 1-, 3-, 5-year BCR-free survival of PCa patients. TCGA, the Cancer Genome Atlas; PSA, prostate specific antigen; SMS, surgical margin status; pT, pathological T stage; GGS, Gleason grade score; BCR, biochemical recurrence.

**FIGURE 8:**
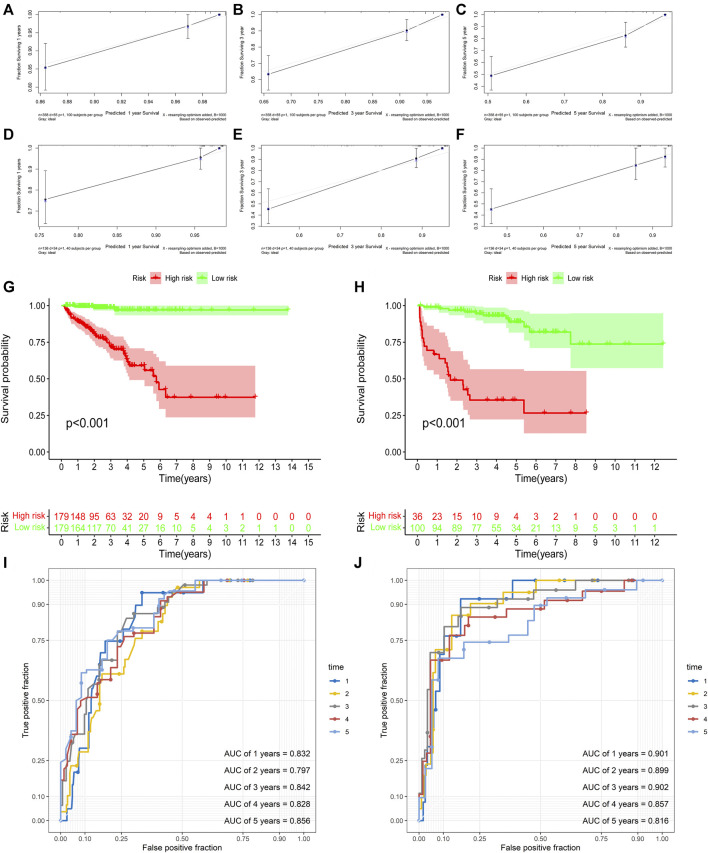
Validation of the nomogram. **(A**–**C)** the calibration curve of the nomogram for predicting 1-, 3-, 5-year BCR of the PCa patients in the TCGA dataset. **(D**–**F)** the calibration curve of the nomogram for predicting 1-, 3-, 5-year BCR of the PCa patients in GSE21034 dataset. **(G)** Kaplan-Meier curve analysis based the nomogram in the TCGA dataset. **(H)** Kaplan-Meier curve analysis based the nomogram in GSE21034 dataset. **(I)** ROC curve analysis for predicting 1-, 2-, 3-, 4-, and5-year BCR of the PCa patients in the TCGA dataset. **(J)** ROC curve analysis for predicting 1-, 2-, 3-, 4-, and 5-year BCR of the PCa patients in GSE21034 dataset. BCR, biochemical recurrence; PCa, prostate cancer; TCGA, the Cancer Genome Atlas; AUC, area under ROC curve; ROC, receiver operating characteristic.

First, univariate Cox analysis and multivariate Cox analysis were applied to evaluate the prognostic significance of the URGs signature combined with different clinicopathological parameters in the TCGA dataset (Figures 7A,B). Next, a nomogram was constructed to quantitatively predict the prognosis of PCa patients based on clinicopathological parameters and the URGs signature. Age was excluded because of the insignificant prognostic value (*p* = 0.995) in univariate Cox analysis. Then, PSA, SMS, GGS, and pT and URGs signature were enrolled to constructed the nomogram (Figure 7C). The calibration curve showed good consistency between the predicted results and the actual results in both the TCGA dataset and the GSE21034 dataset (Figures 8A–F). Using the median risk score of TCGA nomogram model as the cut-off value, the patients were stratified to high-risk and low-risk groups in both the TCGA dataset and GSE21034 dataset. The results of Kaplan-Meier curve analysis showed that patients of high-risk group had poorer BCR-free survival prognosis in both the TCGA dataset and GSE21034 dataset (Figures 8G,H). In TCGA dataset, the AUC values for 1-, 2-, 3-, 4-, and 5-year BCR survival were 0.832, 0.797, 0.842, 0.828, and 0.856, respectively (Figure 8I) and the C index was 0.810 (95% CI: 0.766–0.853). In the GSE21034 dataset, the AUC values were 0.901, 0.899, 0.902, 0.857, and 0.816 at 1-, 2-, 3-, 4, and 5-year, respectively (Figure 8J), and C index was 0.854 (95% CI: 0.799–0.910).

## Discussion

BCR will occur in a sizeable proportion of patients with localized PCa after radical prostatectomy (Liesenfeld et al., 2017). Early BCR is associated with high risk of recurrence and metastasis of PCa. Therefore, an effective method should be established to early predict BCR to improve the prognosis of PCa patients. It is important to identify the patients with high-risk of BCR.

In our study, we constructed a gene signature based on URGs. These genes included ARIH2, FBXO6, GNB4, HECW2, LZTR1, and RNF185. ARIH2 is a RING-in-between-RING E3 ligase gene, its encoding protein has tumor suppressive function and also involves in the neuronal response to hypoxia (Wang et al., 2020). FBXO6 is a member of the F-box protein family which is characterized by a 40 amino acid motif, F-box. The protein encoded by this gene may participate in the regulation of the cell cycle (Wu et al., 2017). FBXO6 protein can promote the growth and proliferation of gastric cancer cells and normal gastric cells (Zhang et al., 2009). Guanine nucleotide-binding protein subunit beta-4 (GNB4) is an important component of heterotrimeric G protein, which transmits signal from G protein-coupled receptors to downstream pathways. It can participant in regulating various biological behaviors of both normal and tumor cells (Gao et al., 2020). Study has showed that GNB4 promotes the tumor progression and chemoresistance in breast cancer, and the high expression of this gene is associated with worse survival rate of colorectal cancer (Riemann et al., 2009; Wang et al., 2018). HECW2 is a HECT-type E3 ubiquitin ligase belonging to the NEDD4 family. The biological function of HECW2 protein is to regulate ubiquitination and stabilize tumor suppressor p73 (Miyazaki et al., 2003). HECW2 also functions as a mediator of proteasomal degradation of DNA damage checkpoint signaling kinase, ATR, in lamin-misexpressing cells (Lu et al., 2013). LZTR1 encodes Golgi protein belonging to the BTB-Kelch superfamily and may be participated in apoptosis and ubiquitination (Nacak et al., 2006). It is also known as a tumor suppressor and the germline and somatic mutations of this gene are associated with schwannomatosis and glioblastoma (Piotrowski et al., 2014) (Franceschi et al., 2016). RNF185 encodes an E3 ubiquitin ligase. RNF185 protein can impact the degradation of BNIP1 and Dvl2, which induce autophagy and osteogenesis, respectively (Tang et al., 2011; Zhou et al., 2014). In addition, the expression levels of RNF185 are positively associated with the lymph node and distant metastasis in renal cell carcinomas patients (de Martino et al., 2012).

According to the survival and ROC curve analysis of TCGA dataset and GSE21034 dataset, this URGs signature had a good diagnostic ability and could be used to identify the PCa patients with poor prognosis of BCR. In addition, the URGs signature could also predict the BCR-free survival of PCa patients in different clinicopathological stratifications and the signature was significantly associated with advanced clinical stage and pathological grade. Finally, a nomogram was established to provide a straightforward and convenient scoring system and help clinical decision making.

To the best of our knowledge, a prognostic model based on URGs and the associated nomogram in PCa has not been reported yet. This model had a good predictive performance and could help identify the patients with high risk of recurrence and make treatment decision. However, there are also some limitations. First, most of the patients in training and validation dataset were from North America, so it is controversial to apply this model to other ethnicities. Second, the construction and validation of the model was designed by retrospective analysis and prospective clinical study should be conducted to validate the model. Finally, the exact molecular mechanisms and biological functions of the URGs should be further investigated.

## Conclusion

We systematically analyzed the prognostic value of URGs and constructed a prognostic model in PCa by bioinformatics techniques. This URGs signature was an independent prognostic factor for predicting the BCR-free survival of PCa patients. A nomogram combining clinicopathological parameters and this signature would be useful to identify the patients with high risk of BCR.

## Data Availability

The original contributions presented in the study are included in the article/Supplementary Material, further inquiries can be directed to the corresponding authors.
